# The socioeconomic burden of antibiotic resistance in conflict-affected settings and refugee hosting countries: a systematic scoping review

**DOI:** 10.1186/s13031-021-00357-6

**Published:** 2021-04-06

**Authors:** Elsa Kobeissi, Marilyne Menassa, Krystel Moussally, Ernestina Repetto, Ismail Soboh, Marwan Hajjar, Shadi Saleh, Ghassan Abu-Sittah

**Affiliations:** 1 Conflict Medicine Program, Global Health Institute, American University of Beirut, Beirut, Lebanon; 2 Lebanon Branch Office, Médecins Sans Frontières, Beirut, Lebanon; 3 Medical Department, Operational Center Brussels, Médecins Sans Frontières, Brussels, Belgium; 4 Division of Plastic Surgery, Department of Surgery, American University of Beirut Medical Center, P.O. Box 11-0236, Riad El Solh, Beirut, 1107-2020 Lebanon; 5 Global Health Institute, American University of Beirut, Beirut, Lebanon

**Keywords:** Antibiotic resistance, Multi-drug resistance, Conflict-affected, Refugee, Socioeconomic, Cost of illness

## Abstract

**Background:**

Antibiotic resistance (ABR) is a major global threat. Armed and protracted conflicts act as multipliers of infection and ABR, thus leading to increased healthcare and societal costs. We aimed to understand and describe the socioeconomic burden of ABR in conflict-affected settings and refugee hosting countries by conducting a systematic scoping review.

**Methods:**

A systematic search of PubMed, Medline (Ovid), Embase, Web of Science, SCOPUS and Open Grey databases was conducted to identify all relevant human studies published between January 1990 and August 2019. An updated search was also conducted in April 2020 using Medline/Ovid. Independent screenings of titles/abstracts followed by full texts were performed using pre-defined criteria. The Newcastle-Ottawa Scale was used to assess study quality. Data extraction and analysis were based on the PICOS framework and following the PRISMA-ScR guideline.

**Results:**

The search yielded 8 studies (7 publications), most of which were single-country, mono-center and retrospective studies. The studies were conducted in Lebanon (*n* = 3), Iraq (*n* = 2), Jordan (*n* = 1), Palestine (n = 1) and Yemen (n = 1). Most of the studies did not have a primary aim to assess the socioeconomic impact of ABR and were small studies with limited statistical power that could not demonstrate significant associations. The included studies lacked sufficient information for the accurate evaluation of the cost incurred by antibiotic resistant infections in conflict-affected countries.

**Conclusion:**

This review highlights the scarcity of research on the socioeconomic burden of ABR on general populations in conflict-affected settings and on refugees and migrants in host countries, and lists recommendations for consideration in future studies. Further studies are needed to understand the cost of ABR in these settings to develop and implement adaptable policies.

**Supplementary Information:**

The online version contains supplementary material available at 10.1186/s13031-021-00357-6.

## Background

Antibiotic resistance (ABR) and multi-drug resistance (MDR) are major global threats [1]. They result in millions of serious infections and in thousands of deaths and disabilities each year [2, 3]. Studies from high income countries show that ABR infections are associated with higher medical costs, prolonged hospital stays, and increased mortality. In the United States alone, ABR accounts for $20 billion in excess direct costs, such as healthcare expenses, and $35 billion in societal costs, such as loss in productivity, annually [2]. In the European Union, about 1.4 billion Euros are spent yearly due to resistance, which is expected to result in 569 million extra hospital days annually by 2050 [3]. This is more problematic in Fragile and Conflict-Affected Situations (FCAS) that are primarily low and middle-income countries (LMIC), where medical care is mostly privatized and costs are often out-of-pocket or paid by third-party insurers [4].

According to a study conducted in Baghdad, disability due to conflict-related injuries affected more than half of injured civilians, most of which were caused by blasts and gunshot wounds [5]. These types of injuries are associated with increased pain intensity and decreased physical function and ability to participate in social roles and activities [6], which further increase the costs on the patients, healthcare system, and society [7]. A study evaluating the economic burden of traumatic injuries in Haiti showed that gunshot wounds had the highest total mean costs, compared to other traumatic injuries [8]. In addition to the clinical and socioeconomic burden caused by the injuries themselves, conflict-related injuries are at high risk of resistant infection due to large tissue defects and environmental contamination, which incur additional costs on patients and hospitals [9]. It is therefore imperative to determine the cost burden to patients and third-party payers when considering the high rates of resistance frequently associated with multiple life-threatening conditions in frail populations in these settings [10]. It is also important to assess the burden to health systems as budgets allocated to healthcare are limited and emergency funds need to be accounted for annually for more urgent responses directly resulting from conflict [11].

Previously implemented interventions in regular settings, based on economic evaluations, have proven to be cost-effective in reducing the clinical and socioeconomic burden of ABR, such as the hospital wide intervention program implemented in Argentina to optimize antibiotic prescribing. Findings have shown that the program decreased resistance to certain antibiotics and contributed to substantial cost savings [12].

The existing literature on the economic burden of ABR mostly consists of studies conducted in regular settings presenting inconsistent results. Several previous systematic reviews, of mostly retrospective and case-control studies, concluded that ABR is usually associated with significantly higher economic burden; however, none were specific to conflict settings [13–17]. Economic evaluations have also thus far focused on healthcare costs without attempting to measure the broader societal cost of ABR and its associated health burden [10]. Due to the complexity of these evaluations, there is no current consensus on methods of estimating the economic burden of ABR [18].

In view of the inconsistent literature related to the socioeconomic burden of ABR in conflict settings, a systematic scoping review was conducted with the objective to answer the following research question: What is the socioeconomic burden of ABR in FCAS and in LMIC that are hosting refugee populations - hereinafter referred to as Refugee Hosting Countries (RHC) - compared to those with susceptible infections/colonization or control patients without infection/colonization? The aims of this review are: 1) to identify the available studies and the gaps in literature on the socioeconomic burden of ABR in conflict settings, 2) to assess the socioeconomic burden of ABR in FCAS and RHC, and 3) to raise awareness about the urgency of the problem and inform policy for the development and implementation of cost-effective interventions tackling ABR in these settings. To the best of our knowledge, this has not been done before and could provide valuable insights and guidance for future work in this area.

## Methods

The study design and analysis were conducted in accordance with the Preferred Reporting Items for Systematic reviews and Meta-Analyses extension for Scoping Reviews (PRISMA-ScR) guidelines (see Appendix I in Additional file 2).

### Search strategy

The search strategy was designed with the help of a library specialist. A search of the PubMed, Medline (Ovid), Embase, Web of Science, SCOPUS and Open Grey databases was performed to identify relevant studies published between January 1990 and August 2019. An updated search was conducted on April the 6th, 2020 using Medline/Ovid. Additional searches were performed using Google Scholar and a manual search of citations of retrieved studies to identify articles that were missed by electronic search. Identified articles were imported into EndNote where duplicates were removed. A full description of the search terms and search strategy is provided in Appendix II (see Additional file 2).

### Selection criteria

Inclusion and exclusion criteria were predefined according to the PICOS framework (see Table 1). The review included articles written in either English, French or Arabic and accompanied by an English abstract.

**Table 1:** Inclusion/exclusion criteria applied

Criteria	Inclusion	Exclusion
Population	Humans	Animals/Plants
	All ages/sexes	
	Individuals in FCAS^a^	
	Refugees or migrants in LMIC^a^	
Intervention/Exposure	Infections/colonization with an antibiotic resistant organism (*Acinetobacter baumannii*, *Pseudomonas aeruginosa*, *Enterobacteriaceae* (*Escherichia coli* and/or *Klebsiella pneumonia*), and/or *Staphylococcus aureus*)	Tuberculosis
Comparator	Susceptible infections/colonization or absence of infection/colonization	
Outcomes	Associated health burden (mortality, morbidity …)	Molecular biology only
	All economic perspectives	
	Associated healthcare cost burden, direct costs (resource use, opportunity cost …)	Epidemiology only
	Indirect costs (loss of productivity …)	
	Intangible costs (decreased quality of life …)	
Study design	Case-control studies	Conference abstracts
	Cohort studies	Posters
	Cross-sectional studies	Systematic reviews
	Randomised controlled studies	
	Modelling studies	
	Economic evaluations	

^a^*FCAS* Fragile and Conflict-Affected Situations; *LMIC* Low- and Middle-Income Countries

Studies published before 1990 were not considered to ensure that the analysis focuses on contemporary literature that reflects relatively recent resistance patterns, economic and financial situations, and clinical practice guidelines. Studies on drug resistant tuberculosis were excluded as we believe they should be analyzed separately due to the bacteria’s specificities and the complexity of multi-drug resistant tuberculosis, its health burden (including treatment) and therefore its financial burden; more importantly the studies were excluded as this microorganism is not specific to conflict-affected areas, subject of this review. Conference abstracts and posters were also excluded as 1) they are largely driven by their brevity and 2) their dependability is questionable.

### Adopted definitions

Several definitions of resistance patterns exist and differ between studies. Therefore, we summarized the details on diagnostic tests and definitions of resistance/multi-drug resistance in Table S1 (see Additional file 1). FCAS and LMIC were defined as per the World Bank definitions [19, 20]. The list of countries included following these definitions are listed under Appendix III (see Additional file 2). The socioeconomic costs of ABR, assessed by conducting cost of illness (COI) studies, are measured by converting the burden associated with ABR in the given society into economic and monetary values. The components of costs include direct costs, indirect costs and intangible costs. Direct costs refer to expenses paid for the treatment and management of illnesses. They combine direct healthcare costs, such as hospitalization and medication costs paid at medical institutions and dispensaries, and direct non-healthcare costs, such as costs of transportation and caregiving. Indirect costs refer to losses of labor and productivity incurred by the illness, such as absenteeism from work and losses of leisure time. Intangible costs refer to costs associated with declines in quality of life and psychological suffering caused by the illness [21].

### Study selection

Identified records were initially screened for their relevance based on titles and abstracts. Articles were screened using the question: “Does this citation potentially describe the socioeconomic burden of antibiotic resistance in FCAS and RHC listed in the protocol?” Full texts of eligible articles were then retrieved and reviewed for inclusion. Articles were independently screened by two reviewers and uncertainty was resolved through discussion with a third author.

### Data extraction and synthesis

We developed a standardized extraction form to record the characteristics of each study and major contributing factors to hospital and patients’ costs. Extraction information included first author, publication year, study period, study location, study design, study population, definitions of cases and controls, patient characteristics, hospital ward, source of infection, microorganisms studied, antibiotic drugs studied, ABR/MDR prevalence, length of stay, number of procedures performed, amputation, mortality, other outcomes, direct costs and indirect costs, as well as any other costs. The form was based on the PICOS framework and was tested on one of the articles to ensure that it covers all relevant information. Data extraction was conducted independently by two researchers and disagreements were resolved by a third reviewer. When relevant data was not published, authors were contacted for additional information. The summary of findings describes the study characteristics and reports the number of studies examining each of the variables considered. The findings describe the direction of the associations and the mean and percentage ranges, whenever possible.

### Study quality assessment

Study quality was evaluated independently by two authors using the Newcastle–Ottawa Scale (NOS), a tool to assess the quality of non-randomized studies (see Appendix IV in Additional file 2). The tool consists of a “star system” to judge the study on three broad perspectives: (1) selection population; (2) comparability of the groups; and (3) ascertainment of either the exposure or outcome of interest. We split the scale into three categories with scores of 0–3, 4–6 and 7–9 representing low, medium and high-quality studies respectively. A consensus was reached on a final range of scores for included studies whenever there was a disagreement. Several assumptions were made to allow for the assessment of all types of observational studies.

## Results

**Fig. 1:**
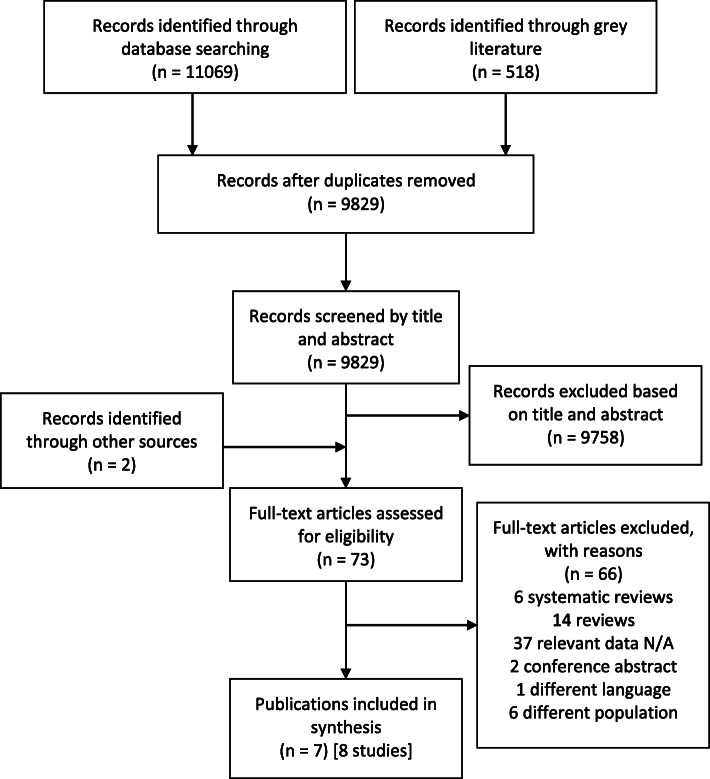
Study flow diagram in accordance with PRISMA statement with modifications

A total of 11,587 articles were identified; 3384 from Embase, 3221 from Medline/Ovid, 2393 from Scopus, 1207 from Web of Science, 864 from PubMed, and 518 from Open Grey. None were identified from the updated search. After removal of duplicates, 9829 articles remained. Out of this total, 9758 articles were excluded based on titles and abstracts. Two articles were then added based on a manual search of Google Scholar and reference lists of identified studies. The full-text screening of the articles excluded a further 66 articles for reasons reported in Fig. 1. A total of 8 eligible studies from 7 publications were included in the analysis (see Fig. 1). In the overall quality assessment, four studies scored high, three scored medium, and one scored low. Out of 138 LMIC, which incorporate 37 FCAS, five countries were included in our analysis.

### Study characteristics

Three studies were conducted in Lebanon (Beirut and Byblos) [22, 23], two in Iraq (Baghdad and Kurdistan) [24, 25], one in Jordan (Ar Ramtha) [26], one in Palestine (Gaza) [27] and one in Yemen (Sana’a) [28]. The study population (total of 5833 participants) was composed of: 1- Syrian refugees with acute conflict injuries [26], 2- military and civilian populations with conflict-inflicted injuries [24], and 3- either civilians or undetermined populations in the five remaining studies. The results consisted of three retrospective chart reviews [23–25], one cross-sectional study [28], one case-control study [27], one nested case-control [22] and two prospective cohorts [22, 26]. All studies were hospital-based but only three were multicenter, and half of the studies were conducted in intensive care units with or without including other hospital wards [22, 24, 27]. Five studies investigated the burden of only one bacterial species [22, 23, 27, 28]. The most studied organism was *Acinetobacter baumanii* (*n* = 5), followed by *Staphylococcus aureus* (*n* = 4), and *Escherichia coli* (*n* = 3). Six studies reported the prevalence of MDR infections which ranged between 37.5 and 88%. However, none of the studies explicitly and comprehensively assessed the COI from resistant infections. Detailed characteristics of the studies included in this review are summarized in Table S2 (see Additional file 1).

### Resource consumption

#### Hospitalization costs

Only one study estimated the average cost of hospitalization, direct cost, due to resistant infections [22]. In this study, the average cost of hospitalization in the ICU due to MDR *Acinetobacter* infection was estimated to be around $1750 per day. The length of stay (LOS) was reported to increase by up to 2 weeks when patients were diagnosed with device-associated infections caused by the resistant organism, leading to an increase in hospitalization cost by $24,000 for every MDR *Acinetobacter* infection.

#### Length of hospitalization

Two studies reported higher LOS for patients with resistant infections [22, 24]; however, results were not statistically significant. One study inferred the opposite relationship but also with non-statistically significant results [26]. Two studies did not compare results between patients with resistant infections and comparator groups [23, 28], two studies mentioned a prolonged hospitalization without showing the duration [22, 25], and one study did not address the outcome [27]. These studies have reported and compared mean LOS between exposure and comparator groups without estimating the attributable cost due to excess LOS, which contribute to direct and indirect costs.

#### Antibacterial medication use

Three studies investigated the suitability of the treatments provided to infected patients. The studies assessed the effectiveness of the medications prescribed to treat *Acinetobacter baumanii* [27], Methicillin-Resistant *Staphylococcus Aureus* (MRSA) [23], and Extended-Spectrum Beta-Lactamases (ESBL) *Escherichia coli* [28]. Inappropriate treatment was estimated to have occurred in between 53 and 63% of cases. One of the studies found that MDR infections and inadequate antibiotic treatment were associated with increased mortality [27].

#### Number of procedures

One study evaluated the average number of procedures per patient [26], which contributes to direct costs. The authors reported that patients with MDR infections needed, on average, slightly more surgeries than patients with non-MDR infections; however, results were not statistically significant.

### Health outcomes

#### Amputation

One study assessed the risk of amputation in the different groups [26], which entails direct, indirect and intangible costs through increased hospitalization, decreased mobility and quality of life, loss of employment, and prosthesis and rehabilitation services usage. Patients with MDR infections had an 83% higher risk of amputation than patients with non-MDR infections; however, results were statistically non-significant (95% CI 0.34–9.89).

#### Mortality

Three studies reported higher mortality in patients with resistant infections [22, 26, 27]. Two studies did not compare results to comparator groups [22, 25], and three studies did not address the outcome [23, 24, 28]. Studies that assessed the association did not find statistical significance.

## Discussion

To the best of our knowledge, this is the first scoping review aiming at mapping and identifying the evidence regarding the socioeconomic burden of ABR/MDR infections compared to susceptible cases or those without infection or colonization in FCAS and RHCs. There were no studies identified with a primary aim to evaluate the COI of antibiotic resistance, was it from the perspective of the patient, payer or provider. This highlighted the gap in the available evidence on the socioeconomic burden of ABR in those settings. In fact, the identified studies rather assessed the clinical burden of certain infections. In these cases, the evaluation of bacterial resistance and its resulting outcomes and resource consumption were sub-analyses. Subsequently, the sample size for the populations of interest became very small and the results lacked statistical power. When an economic analysis was conducted, a hospital perspective was adopted without taking into account the indirect costs of ABR. LOS, a key driver of cost of infections in hospitals [29], was one of the main outcomes assessed in the identified studies. Although LOS can be used to estimate the number of working days lost [21], the identified studies did not use this indicator to calculate indirect costs of ABR.

Results were not consistent between studies that investigated the difference in LOS of patients with resistant and comparator groups. In addition, the methodology used for this assessment also varied. Studies addressing LOS did not explicitly estimate attributable LOS, but rather the average LOS for the different groups. Studies also did not address the increased risk of death due to resistant infections, but rather reported the prevalence of mortality in the different groups. As for other settings, a study conducted in Australia reported that vancomycin resistance was associated with increased LOS and hospitalization costs [30]. A systematic review on the clinical and economic burden of ABR in developing countries found an association between ABR and high mortality risk [14]. However, another review reported conflicting results, nonetheless concluding that ABR is usually associated with a significant financial burden [17]. Although statistically insignificant, there was weak evidence indicating increased risk of amputation, which would incur indirect costs [31]. Further studies with different methodologies are therefore needed to investigate these associations. Studies that addressed antibiotic use found that treatment of susceptible and resistant infections was inappropriate in more than half the cases and was associated with higher mortality. This is consistent with previous systematic reviews and meta-analyses indicating that improper antibiotic therapies are correlated with increased mortality as well as direct and indirect costs [14, 15].

Previous studies have led to the establishment of cost-effective policies that promote appropriate use of antimicrobials and prevent the spread of infections. Examples include the improvement of hand hygiene strategies which reduce LOS, number of deaths, and total costs [32], and the implementation of antibiotic stewardship programs which decrease LOS and antibiotic expenditure, and increase cost-savings for healthcare systems [16, 33]. Although knowledge of the socioeconomic impact of ABR is needed to influence programs in healthcare facilities and to guide policymakers and funding agencies, data on the cost of ABR in conflict-affected areas are scarce. Further studies are needed to obtain more precise estimates of ABR burden to inform policy through cost-effectiveness or resource allocation models [18]. The comprehensive assessment of the socioeconomic burden of ABR is complex and cannot be easily performed. This is due to the fact that ABR, in economic terminology, is an externality considering that the effect of antibiotic use ultimately impacts the overall welfare of the community. It varies between countries and largely depends on the type of prevalent resistant organisms in different contexts. The challenges of this assessment include the need for a detailed analysis taking into account the specificity of each microorganism in terms of resistance patterns, treatment procedures and associated costs. ABR also impacts the treatment of other diseases as well as the social and economic sectors, such as the labor market, therefore increasing indirect costs [31]. Hence, there is a need for a context-specific analysis of the socioeconomic burden of ABR. Relying on studies conducted in controlled settings would not be reasonable as they are not generalizable to or feasible in conflict settings. In armed and protracted conflicts, medical personnel are overwhelmed by mass casualties or the burden of conflict and the availability of and the accessibility to healthcare facilities and supplies is usually limited in these settings [34]. Patients are more likely to self-diagnose and self-medicate [35] and healthcare professionals are more likely to dispense inappropriate medications; consequently, increasing the risk of ABR development as well as leading to higher costs [34]. Accordingly, conflicts pose additional challenges to health personnel and researchers seeking to establish the socioeconomic burden of ABR in that context; therefore, obstructing the opportunity to build solid evidence to support best practices in emergency situations. As shown by this review, when feasible, the quality of the evidence regarding the socioeconomic impact of ABR is of low quality. The clinical and socioeconomic burden of ABR must be well understood to be able to develop and implement adaptable policies.

Researchers and health professionals play a role in providing evidence on the burden of ABR and in ensuring stakeholders take account of that evidence when developing policies [36]. Based on the results of this review, we provide the following recommendations for future research on ABR and its socioeconomic impact in conflict-affected settings:

- Despite the complexity of conflict-affected settings, it is essential that future studies ensure that definitions are clearly and accurately stated and that samples are representative to allow for standardization and better comparison and synthesis of the evidence to potentially generate reliable evidence and better understanding of conflict-affected settings;
- Data collection might not be a priority in these settings, therefore it needs to be consciously accounted for to generate relative risks or attributable risks of morbidity and mortality from ABR and excess LOS, ICU/hospital stay/cost, and antibiotic and other resource use/cost, whenever possible;
- It is important to consider the confounding effect of relevant covariates and to control for them. Building capacities of local researchers working in conflict-affected settings would improve the quality of studies conducted in these regions. The Structured Operational Research Training Initiative (SORT-IT), implemented and run by Médecins Sans Frontières (MSF) and the Union, and the Center for Research and Education in the Ecology of War (CREEW), run by the Global Health Institute at the American University of Beirut (AUB), are some examples of established programs aiming at equipping frontline health practitioners with the necessary skills to conduct research;
- It would be useful to adopt the conceptual framework for capacity strengthening of health research in conflict and adapting it to conduct economic evaluations in this setting, and advocating for translating research outcomes into services and policies [37].

We conducted a comprehensive search of the literature in multiple databases using the PRISMA guidelines and we have identified the gaps and areas for future research. However, our study was subject to certain limitations. First, as we only included articles with English abstracts, potential language bias cannot be neglected. However, we did not limit our search to only English articles which could mean that the impact on the findings is negligible. Second, although the health burden of ABR has been previously associated with increased costs, most of the included studies in our review did not focus on estimating the excess cost attributed to resistant infections, preventing us from providing a clear estimate of the socioeconomic burden of ABR. Finally, the studies had a great deal of missing information, either due to the retrospective nature of most of the studies or to the emergency context in which the research studies were conducted, thus restricting the proper assessment of the socioeconomic burden of ABR in conflict-affected settings. Given the lack of relevant data, the results should be interpreted with caution.

## Conclusion

There is some evidence indicating that inappropriate antibiotic therapy leads to more resistant infections which is associated with prolonged hospital stay and higher mortality rates in conflict-affected regions. This review emphasizes the lack of studies addressing the socioeconomic burden of ABR in FCAS and RHC, partly due to lack of focused research on this topic and consequently data scarcity. It also highlights the need for well-designed longitudinal or large retrospective multicenter studies examining this relationship and accounting for all the variables that are intrinsic to the nature of conducting research in such challenging settings.

## Supplementary Information

**Additional file 1: Table S1.** Definitions: identification of organisms and susceptibility testing. Details on the microorganisms studied in each of the included studies, the diagnostic tests and the definitions of resistance/multi-drug resistance. **Table S2.** Characteristics of studies investigating the socioeconomic burden of ABR in conflict-affected countries. Characteristics of the studies included in the scoping review. The extracted data includes the first author, publication year, country of origin, center type and number of centers, total population, number of cases and comparison groups, population of interest if different from the total population, number of cases and comparison groups in the subsample, micro-organisms studies, infection and ABR/MDR prevalence, and key findings.

**Additional file 2: Appendix I.** Preferred Reporting Items for Systematic reviews and Meta-Analyses extension for Scoping Reviews (PRISMA-ScR) Checklist. **Appendix II.** Medline/Ovid Search Strategy. Detailed search strategy for one of the databases used to conduct this scoping review. **Appendix III.** Categorization of countries according to the World Bank Data. “Fragile and conflict affected situations” and “Low & middle income”. **Appendix IV.** Quality assessment checklists. The Newcastle-Ottawa Scale to assess the quality of studies. Appendix IV. a. includes the checklist for all studies excluding cohort studies, and Appendix IV. b. includes the checklist for cohort studies.

## Data Availability

All data generated or analysed during this study are included in this published article and its supplementary information files.

## Abbreviations

ABR: Antibiotic Resistance
AUB: American University of Beirut
COI: Cost of Illness
CREEW: Center for Research and Education in the Ecology of War
ESBL: Extended Spectrum Beta-Lactamase
FCAS: Fragile and Conflict Affected Situations
ICU: Intensive Care Unit
LMIC: Low and Middle Income Countries
LOS: Length of Stay
MDR: Multidrug Resistance
MRSA: Methicillin-Resistant *Staphylococcus aureus*
MSF: Médecins Sans Frontières
NOS: Newcastle-Ottawa Scale
PICOS: Population, Intervention, Comparison, Outcome, Study type
PRISMA-ScR: Preferred Reporting Items for Systematic reviews and Meta-Analyses extension for Scoping Reviews
RHC: Refugee Hosting Country

## References

[1] World Health Organization. Antimicrobial resistance 2018 [cited 2019 2019/05/30]. Available from: https://www.who.int/en/news-room/fact-sheets/detail/antimicrobial-resistance.

[2] Centers for Disease Control and Prevention. Antibiotic resistance threats in the United States, 2013. 2013 [cited 2020 22 April, 2020]. Available from: https://www.cdc.gov/drugresistance/pdf/ar-threats-2013-508.pdf.

[3] European Centers for Disease Control and Prevention. Antimicrobial resistance: trackling the burden in the European Union. 2019. [cited 2020 22 April, 2020]. Available from: https://www.oecd.org/health/health-systems/AMR-Tackling-the-Burden-in-the-EU-OECD-ECDC-Briefing-Note-2019.pdf.

[4] World Health Organization. Health financing policy and implementation in fragile and conflict-affected settings: Synthesis of evidence and policy recommendations. 2019 Contract No.: WHO/HIS/HGF/HFGuidance/19.7.

[5] Lafta R, Al-Shatari S, Cherewick M, Galway L, Mock C, Hagopian A (2015). Injuries, death, and disability associated with 11 years of conflict in Baghdad, Iraq: a randomized household cluster survey. PLoS One.

[6] Vella MA, Warshauer A, Tortorello G, Fernandez-Moure J, Giacolone J, Chen B, Cabulong A, Chreiman K, Sims C, Schwab CW, Reilly PM, Lane-Fall M, Seamon MJ (2020). Long-term functional, psychological, emotional, and social outcomes in survivors of firearm injuries. JAMA Surg.

[7] Edwards DS, Phillip RD, Bosanquet N, Bull AMJ, Clasper JC (2015). What Is the Magnitude and Long-term Economic Cost of Care of the British Military Afghanistan Amputee Cohort?. Clinical Orthopaedics and Related Research®.

[8] Zuraik C, Sampalis J, Brierre A (2018). The economic and social burden of traumatic injuries: evidence from a trauma Hospital in Port-au-Prince, Haiti. World J Surg.

[9] Sahli ZT, Bizri AR, Abu-Sittah GS (2016). Microbiology and risk factors associated with war-related wound infections in the Middle East. Epidemiol Infect.

[10] Gandra S, Barter DM, Laxminarayan R (2014). Economic burden of antibiotic resistance: how much do we really know?. Clin Microbiol Infect.

[11] Debarre A. Hard to reach: providing healthcare in armed conflict. International peace institute., December 2018. Report No.

[12] Bantar C, Sartori B, Vesco E, Heft C, Saúl M, Salamone F (2003). A Hospitalwide intervention program to optimize the quality of antibiotic use: impact on prescribing practice, antibiotic consumption, cost savings, and bacterial resistance. Clin Infect Dis.

[13] Alsan M, Schoemaker L, Eggleston K, Kammili N, Kolli P, Bhattacharya J (2015). Out-of-pocket health expenditures and antimicrobial resistance in low-income and middle-income countries: an economic analysis. Lancet Infect Dis.

[14] Founou RC, Founou LL, Essack SY (2017). Clinical and economic impact of antibiotic resistance in developing countries: A systematic review and meta-analysis. PLoS ONE [Electronic Resource].

[15] Nathwani D, Raman G, Sulham K, Gavaghan M, Menon V (2014). Clinical and economic consequences of hospital-acquired resistant and multidrug-resistant Pseudomonas aeruginosa infections: A systematic review and meta-analysis. Antimicrobial Resistance and Infection Control.

[16] Nathwani D, Varghese D, Stephens J, Ansari W, Martin S, Charbonneau C (2019). Value of hospital antimicrobial stewardship programs [ASPs]: a systematic review. Antimicrobial Resistance Infection Control.

[17] Zhen X, Lundborg CS, Sun X, Hu X, Dong H. Economic burden of antibiotic resistance in ESKAPE organisms: A systematic review. Antimicrobial Resistance and Infection Control. 2019;8(1). 10.1186/s13756-019-0590-7 PMC6692939 31417673

[18] Naylor NR, Atun R, Zhu N, Kulasabanathan K, Silva S, Chatterjee A, Knight GM, Robotham JV (2018). Estimating the burden of antimicrobial resistance: a systematic literature review. Antimicrobial Resistance Infection Control..

[19] Fragile and conflict affected situations: The World Bank [cited 2019 November 6, 2019]. Available from: https://data.worldbank.org/region/fragile-and-conflict-affected-situations.

[20] Low & middle income: The World Bank [cited 2019 November 6, 2019]. Available from: https://data.worldbank.org/income-level/low-and-middle-income.

[21] Choi H-J, Lee E-W. Methodology of Estimating Socioeconomic Burden of Disease Using National Health Insurance (NHI) Data. 2019. In: evaluation of health services [internet]. IntechOpen. Available from: https://www.intechopen.com/books/evaluation-of-health-services/methodology-of-estimating-socioeconomic-burden-of-disease-using-national-health-insurance-nhi-data.

[22] Kanafani ZA, Zahreddine N, Tayyar R, Sfeir J, Araj GF, Matar GM, Kanj SS (2018). Multi-drug resistant Acinetobacter species: a seven-year experience from a tertiary care center in Lebanon. Antimicrob Resist Infect Control.

[23] Matar MJ, Moghnieh R, Alothman AF, Althaqafi AO, Alenazi TH, Farahat FM, Corman S, Solem C, Raghubir N, Macahilig C, Haider S, Stephens J (2017). Treatment patterns, resource utilization, and outcomes among hospitalized patients with methicillin-resistant Staphylococcus aureus complicated skin and soft tissue infections in Lebanon and Saudi Arabia. Infection Drug Resistance.

[24] Aldous WK, Co EM (2010). Factors associated with recovery of multidrug-resistant bacteria in a combat support hospital in Iraq. Infection Control Hospital Epidemiology.

[25] Babakir-Mina M, Othman N, Najmuldeen HH, Noori CK, Fatah CF, Perno CF (2012). Antibiotic susceptibility of vancomyin and nitrofurantoin in Staphylococcus aureus isolated from burnt patients in Sulaimaniyah, Iraqi Kurdistan. New Microbiologica.

[26] Alga A, Wong S, Shoaib M, Lundgren K, Giske CG, von Schreeb J (2018). Infection with high proportion of multidrug-resistant bacteria in conflict-related injuries is associated with poor outcomes and excess resource consumption: a cohort study of Syrian patients treated in Jordan. BMC Infect Dis.

[27] Al Jarousha AM, El Jadba AH, Al Afifi AS, El Qouqa IA (2009). Nosocomial multidrug-resistant Acinetobacter baumannii in the neonatal intensive care unit in Gaza City, Palestine. Int J Infect Dis.

[28] Nasher S, Alsharapy S, Al-Madhagi A, Zakham F (2018). Epidemiology of extended-spectrum beta-lactamase producing Escherichia coli from hospital settings in Yemen. J Infection Developing Countries.

[29] Roberts RR, Scott RD, Hota B, Kampe LM, Abbasi F, Schabowski S (2010). Costs attributable to healthcare-acquired infection in hospitalized adults and a comparison of economic methods. Med Care.

[30] Cheah AL, Spelman T, Liew D, Peel T, Howden BP, Spelman D (2013). Enterococcal bacteraemia: factors influencing mortality, length of stay and costs of hospitalization. Clin Microbiol Infect.

[31] Anderson M, Cecchini M, Mossialos E, North J (2020). Challenges to tackling antimicrobial resistance: economic and policy responses: Cambridge University press.

[32] Strategic Research and Innovation Agenda on Antimicrobial Resistance. Stockholm: Joint Programming Initiative on Antimicrobial Resistance 2019.

[33] Soule BM, Memish ZA, Malani PN (2012). Best practices in infection prevention and control: an international perspective: joint commission international.

[34] Haraoui LP, Sparrow A, Sullivan R, Burci GL, Dewachi O, Abu-Sittah G (2019). Armed conflicts and antimicrobial resistance: a deadly convergence. AMR Control.

[35] Bunduki GK, Katembo J-LM, Kamwira IS (2020). Antimicrobial resistance in a war-torn country: lessons learned in the eastern Democratic Republic of the Congo. One Health.

[36] Lomazzi M, Moore M, Johnson A, Balasegaram M, Borisch B (2019). Antimicrobial resistance – moving forward?. BMC Public Health.

[37] El Achi N, Papamichail A, Rizk A, Lindsay H, Menassa M, Abdul-Khalek RA (2019). A conceptual framework for capacity strengthening of health research in conflict: the case of the Middle East and North Africa region. Glob Health.

