# Supplementary material for: The socioeconomic burden of antibiotic resistance in conflict-affected settings and refugee hosting countries: a systematic scoping review
Source: Confl Health. 2021 Apr 6;15:21. doi: 10.1186/s13031-021-00357-6 (PMC8025481; doi:10.1186/s13031-021-00357-6)
Supplement: Supplementary file 1 — Additional file 1: Table S1. Definitions: identification of organisms and susceptibility testing. Details on the microorganisms studied in each of the included studies, the diagnostic tests and the definitions of resistance/multi-drug resistance. Table S2. Characteristics of studies investigating the socioeconomic burden of ABR in conflict-affected countries. Characteristics of the studies included in the scoping review. The extracted data includes the first author, publication year, country of origin, center type and number of centers, total population, number of cases and comparison groups, population of interest if different from the total population, number of cases and comparison groups in the subsample, micro-organisms studies, infection and ABR/MDR prevalence, and key findings. [file 13031_2021_357_MOESM1_ESM.docx]

**Table S1.** Definitions: identification of organisms and susceptibility testing.

| **First Author (publication year)** | **Andreas Älgå (2018)** | **Wade K. Aldous (2010)** | **Muhammed Babakir-Mina (2012)** | **Abdel Moati Kh. Al Jarousha (2009)** | **Madonna J Matar (2017)** | **Samar Nasher (2018)** | **Zeina A. Kanafani (2018)** |
| --- | --- | --- | --- | --- | --- | --- | --- |
| **Micro-organisms studied** | Staphylococcus aureus, Pseudomonas, Klebsiella pneumoniae, Enterobacter, E. coli, Proteus, and Acinetobacter | Staphylococcus aureus, Escherichia coli, Klebsiella pneumoniae, A. baumannii, and Pseudomonas aeruginosa | Staphylococcus aureus | Acinetobacter baumannii | Staphylococcus aureus | Escherichia coli | Acinetobacter species |
| **Susceptibility testing** | Manual disk diffusion or automated using Vitek® 2 technology.  Interpreted according to the guidelines from the Clinical and Laboratory Standards Institute | n/r according to medical records | Kirby-Bauer method | Disk diffusion method on Mueller-Hinton agar plates using calibrated inoculum of the isolates based on McFarland standard | n/r according to medical records | Kirby Bauer Disk Diffusion method.  Screened by phenotypic disk diffusion method for ESBL production, then confirmed by phenotypic double disk synergy test | Disk diffusion method.  Colistin sensitivity testing: VITEC-2 Bio System and disk diffusion.  Interpreted according to the guidelines from the Clinical and Laboratory Standards Institute |
| **Definition of MDR** | Resistance to at least one antibiotic from three or more relevant antibiotic groups | n/r according to medical records |  | Resistant to three antibiotics out of four of the following: ceftazidime, ciprofloxacin, gentamicin, and imipenem | n/r according to medical records |  | Resistant to all tested antimicrobial agents except colistin and tigecycline |

**Table S2.** Characteristics of studies investigating the socioeconomic burden of ABR in conflict-affected countries.

| **First Author (publication year)  Country**  **Study period** | **Center type, number** | **Total population**  **Age (mean or median)**  **Gender (%)** | **Cases (#)** | **Population of interest, subsample** | **Cases for subsample (#)** | **Micro-organisms** | **Infection prevalence** | **ABR/MDR prevalence** | **Key findings** |
| --- | --- | --- | --- | --- | --- | --- | --- | --- | --- |
|  |  |  | **Comparison group(s) (#)** |  | **Comparison group(s) for subsample (#)** |  |  |  |  |
| Wade K. Aldous (2010)  Iraq (Baghdad)  2005-2009 | Hospital, 1 | 1368  (2286 samples)  N/A  Male (77%) | US Military members (860)  Host nation (Iraqis) (508) | n/r | n/r | S. aureus, E. coli, Klebsiella pneumoniae, Acb baumannii, and  P. aeruginosa | n/r | 968/1368 (70.7%) | **LOS:**  2 (if 1 MDR isolate) 10 (if 2 MDR isolates) 27 (if >=3 MDR isolates) |
| Andreas Älgå (2018)  Jordan (Ar Ramtha)  2014-2016 | Hospital, 1 | 457  27 years  Male (86%) | With infection (49)  Without infection (408) | 49 | MDR infection (36)  non-MDR infection (13) | S. aureus, E. coli, Klebsiella pneumoniae, Enterobacter, Proteus, Acb strains, and  P. aeruginosa | 49/457 (11%) - lab verified | 36/49 (73%) | **LOS:**  69 (for MDR), 99 (for non-MDR), p=0.131  **Average # of procedures:** 13 (for MDR), 11 (for non-MDR), p=0.69 **Mortality:** 2 out of 36 (5.5%) for MDR,  0 out of 15 for non-MDR **Amputation**: 25% (for MDR),  15% (for non-MDR),  RR=1.83 (95% CI 0.34–9.89) |
| Muhammed Babakir-Mina (2012)  Iraq (Kurdistan)  2008-2011 | Hospital, 1 | 2938  18 years  Male (42%) | With Staph aureus infection (172)  Without Staph aureus infection (437) | n/r | n/r | S. aureus | 654 (22%) - staph aureus | 575/654 (88%) - MRSA | **LOS:** 17 (median) (with S. aureus infection)  Higher in patients with MRSA infection. **Mortality:**  98 (15%) (with S. aureus infection) |

**Table S2.** (Continued)

| **First Author (publication year)  Country**  **Study period** | **Center type, number** | **Total population**  **Age (mean or median)**  **Gender (%)** | **Cases (#)** | **Population of interest, subsample** | **Cases for subsample (#)** | **Micro-organisms** | **Infection prevalence** | **ABR/MDR prevalence** | **Key findings** |
| --- | --- | --- | --- | --- | --- | --- | --- | --- | --- |
|  |  |  | **Comparison group(s) (#)** |  | **Comparison group(s) for subsample (#)** |  |  |  |  |
| Abdel Moati Kh. Al Jarousha (2009)  Palestine (Gaza)  2004-2005 | Hospital, 2 | 579  N/A  <28 days  Male (62.5%) | Infected with Acb sepsis (40)  1- Non-bloodstream infection (100)  2- Infected with non-Acb sepsis (106) | 40 | MDR Acb (15)  non-MDR Acb (25) | Acb (of interest)/ Others: E. coli, S. aureus, Klebsiella, coagulase-negative Staphylococci, Enterobacter, Streptococcus, Citrobacter, and P. aeruginosa | 40 (6.9%) | 15/40 (37.5%) - MDR Acb | **MDR-Acb prevalence:**  6 (24%) (for survivors)  9 (60%) (for deaths)  p=0.023  **Mortality:**  9/15 (60%) (for MDR Acb), 6/25 (24%) (for non-MDR Acb)  **Inappropriate treatment:**  6 (24%) (for Acb survivors)  8 (53.3%) (for Acb deaths), p=0.01 |
| Zeina A. Kanafani (2018)  Lebanon  2007-2014 | Hospital, 1 | 128 (with Acb)  58 years  Male (60.2%) | With Acb (128) | n/r | n/r | Acb spp. | n/r | 95/128 (74%) | **LOS:** 3.6 (1-14)- overall,  9.7 (1-150) (for MDR Acb) **Mortality:** 28 (22%) - overall  **Hospital costs:** MDR-Ab average cost of stay up to $1750/day |
| Zeina A. Kanafani (2018)  Lebanon  2007-2008 | Hospital, 1 | 146  61.7 years (cases)  60.4 years (controls)  2:1 male to female ratio | Acb infections or colonization (73)  No infection (73) | 73 | MDR Acb infections or colonization (40)  Negative MDR-Acb culture (33) | Acb baumannii complex (96%), other Acb spp. | n/r | 40/73 (55%) | **LOS:** 31/40 (77.5%) (for MDR Acb), 27/33 (81.2%) (for non-MDR Acb) had prolonged hospitalization (LOS not specified) **Mortality:** 22/40 (55%) (for MDR Acb), 12/33 (36.4%) (for non-MDR Acb) |

| **First Author (year)  Country**  **Study period** | **Center type, number** | **Total population**  **Age (mean or median)**  **Gender (%)** | **Cases (#)** | **Population of interest, subsample** | **Cases for subsample (#)** | **Micro-organisms** | **Infection prevalence** | **ABR/MDR prevalence** | **Key results** |
| --- | --- | --- | --- | --- | --- | --- | --- | --- | --- |
|  |  |  | **Comparison group(s) (#)** |  | **Comparison group(s) for subsample (#)** |  |  |  |  |
| Madonna J Matar (2017)  Lebanon, Saudi Arabia  2011-2012 | Hospital, 5 | 87  52 years  Male (61%) | MRSA (no comparison group) | 38 | Lebanese subsample | MRSA | n/r | n/r | **LOS:** 14.5 (for MRSA) **Inactive treatment:**  63% (for MRSA) **Treatment failure:** 5% **Intermediate response:** 5% **Relapse of MRSA:** 5% **Re-hospitalization for MRSA:** 3% |
| Samar Nasher (2018)  Yemen (Sana'a)  2014-2015 | Hospital, 2 | 130  N/A  Most prevalent age range: >50 years  Male (52.3%) | ESBL (44)  Non-ESBL (86) | n/r | n/r | E. coli | n/r | 33.8% (ESBL) | **LOS:** 7 to >=22 days 15-21 days most prevalent range  7-14 days: 18.2%, (for ESBL), OR = 1.71 (0.27-4.70) 15-21 days: 32.1%, (for ESBL), OR = 2.24 (0.34-3.76) ≥ 22 days: 47.7%, (for ESBL), OR = 2.80 (0.36-5.45) p = 0.04  **Inappropriate treatment:**  22 (56.4%) (for ESBL E.coli)  22 (24.2%) (for non-ESBL E.coli) |
| ABR = Antibiotic Resistance; MDR = Multi-Drug Resistance; Acb = Acinetobacter; P. aeruginosa = Pseudomonas aeruginosa; K. pneumonia = Klebsiella pneumonia; E. coli = Escherichia coli; S. aureus = Staphylococcus aureus; MRSA = Methicillin-resistant Staphylococcus aureus; ESBL = Extended spectrum beta-lactamases; n/r = non-reported or non-applicable | | | | | | | | | |

**Table S2.** (Continued)
