# Supplementary material for: The socioeconomic burden of antibiotic resistance in conflict-affected settings and refugee hosting countries: a systematic scoping review
Source: Confl Health. 2021 Apr 6;15:21. doi: 10.1186/s13031-021-00357-6 (PMC8025481; doi:10.1186/s13031-021-00357-6)
Supplement: Supplementary file 2 — Additional file 2: Appendix I. Preferred Reporting Items for Systematic reviews and Meta-Analyses extension for Scoping Reviews (PRISMA-ScR) Checklist. Appendix II. Medline/Ovid Search Strategy. Detailed search strategy for one of the databases used to conduct this scoping review. Appendix III. Categorization of countries according to the World Bank Data. “Fragile and conflict affected situations” and “Low & middle income”. Appendix IV. Quality assessment checklists. The Newcastle-Ottawa Scale to assess the quality of studies. Appendix IV. a. includes the checklist for all studies excluding cohort studies, and Appendix IV. b. includes the checklist for cohort studies. [file 13031_2021_357_MOESM2_ESM.docx]

**Appendices**

**Appendix I.** Preferred Reporting Items for Systematic reviews and Meta-Analyses extension for Scoping Reviews (PRISMA-ScR) Checklist

| **SECTION** | **ITEM** | **PRISMA-ScR CHECKLIST ITEM** | **REPORTED ON PAGE #** |
| --- | --- | --- | --- |
| **TITLE** | | | |
| Title | 1 | Identify the report as a scoping review. | 1 |
| **ABSTRACT** | | | |
| Structured summary | 2 | Provide a structured summary that includes (as applicable): background, objectives, eligibility criteria, sources of evidence, charting methods, results, and conclusions that relate to the review questions and objectives. | 2, 3 |
| **INTRODUCTION** | | | |
| Rationale | 3 | Describe the rationale for the review in the context of what is already known. Explain why the review questions/objectives lend themselves to a scoping review approach. | 3, 4 |
| Objectives | 4 | Provide an explicit statement of the questions and objectives being addressed with reference to their key elements (e.g., population or participants, concepts, and context) or other relevant key elements used to conceptualize the review questions and/or objectives. | 5 |
| **METHODS** | | | |
| Protocol and registration | 5 | Indicate whether a review protocol exists; state if and where it can be accessed (e.g., a Web address); and if available, provide registration information, including the registration number. | Not published |
| Eligibility criteria | 6 | Specify characteristics of the sources of evidence used as eligibility criteria (e.g., years considered, language, and publication status), and provide a rationale. | 6, 7 |
| Information sources* | 7 | Describe all information sources in the search (e.g., databases with dates of coverage and contact with authors to identify additional sources), as well as the date the most recent search was executed. | 5 |
| Search | 8 | Present the full electronic search strategy for at least 1 database, including any limits used, such that it could be repeated. | Appendix II |
| Selection of sources of evidence† | 9 | State the process for selecting sources of evidence (i.e., screening and eligibility) included in the scoping review. | 8 |
| Data charting process‡ | 10 | Describe the methods of charting data from the included sources of evidence (e.g., calibrated forms or forms that have been tested by the team before their use, and whether data charting was done independently or in duplicate) and any processes for obtaining and confirming data from investigators. | 8 |
| Data items | 11 | List and define all variables for which data were sought and any assumptions and simplifications made. | 8 |
| Critical appraisal of individual sources of evidence§ | 12 | If done, provide a rationale for conducting a critical appraisal of included sources of evidence; describe the methods used and how this information was used in any data synthesis (if appropriate). | 9 |
| Synthesis of results | 13 | Describe the methods of handling and summarizing the data that were charted. | 8 |
| **RESULTS** | | | |
| Selection of sources of evidence | 14 | Give numbers of sources of evidence screened, assessed for eligibility, and included in the review, with reasons for exclusions at each stage, ideally using a flow diagram. | 9 |
| Characteristics of sources of evidence | 15 | For each source of evidence, present characteristics for which data were charted and provide the citations. | 10, Table 3 |
| Critical appraisal within sources of evidence | 16 | If done, present data on critical appraisal of included sources of evidence (see item 12). | 9 |
| Results of individual sources of evidence | 17 | For each included source of evidence, present the relevant data that were charted that relate to the review questions and objectives. | Table 3 |
| Synthesis of results | 18 | Summarize and/or present the charting results as they relate to the review questions and objectives. | 10-12 |
| **DISCUSSION** | | | |
| Summary of evidence | 19 | Summarize the main results (including an overview of concepts, themes, and types of evidence available), link to the review questions and objectives, and consider the relevance to key groups. | 12-15 |
| Limitations | 20 | Discuss the limitations of the scoping review process. | 16 |
| Conclusions | 21 | Provide a general interpretation of the results with respect to the review questions and objectives, as well as potential implications and/or next steps. | 16, 17 |
| **FUNDING** | | | |
| Funding | 22 | Describe sources of funding for the included sources of evidence, as well as sources of funding for the scoping review. Describe the role of the funders of the scoping review. | 18 |

JBI = Joanna Briggs Institute; PRISMA-ScR = Preferred Reporting Items for Systematic reviews and Meta-Analyses extension for Scoping Reviews.

* Where *sources of evidence* (see second footnote) are compiled from, such as bibliographic databases, social media platforms, and Web sites.

† A more inclusive/heterogeneous term used to account for the different types of evidence or data sources (e.g., quantitative and/or qualitative research, expert opinion, and policy documents) that may be eligible in a scoping review as opposed to only studies. This is not to be confused with *information sources* (see first footnote).

‡ The frameworks by Arksey and O’Malley (6) and Levac and colleagues (7) and the JBI guidance (4, 5) refer to the process of data extraction in a scoping review as data charting*.*

§ The process of systematically examining research evidence to assess its validity, results, and relevance before using it to inform a decision. This term is used for items 12 and 19 instead of "risk of bias" (which is more applicable to systematic reviews of interventions) to include and acknowledge the various sources of evidence that may be used in a scoping review (e.g., quantitative and/or qualitative research, expert opinion, and policy document).

*From:* Tricco AC, Lillie E, Zarin W, O'Brien KK, Colquhoun H, Levac D, et al. PRISMA Extension for Scoping Reviews (PRISMAScR): Checklist and Explanation. Ann Intern Med. 2018;169:467–473. [doi: 10.7326/M18-0850](http://annals.org/aim/fullarticle/2700389/prisma-extension-scoping-reviews-prisma-scr-checklist-explanation).

**Appendix II.** Medline/Ovid Search Strategy

((Anti-Bacterial Agents/ or Bacterial Infections/) and Drug Resistance/) or exp Drug Resistance, Bacterial/ or Drug Resistance, Microbial/ or Drug Resistance, Multiple/ or Microbial Sensitivity Tests/

((Staphylococcal Infections/ or exp Staphylococcus aureus/ or Enterobacteriaceae/ or Enterobacter aerogenes/ or Enterobacter/ or Escherichia coli/ or Escherichia coli Infections/ or Klebsiella/ or Klebsiella Infections/ or Klebsiella pneumoniae/ or Streptococcal Infections/ or Streptococcaceae/ or Streptococcus/ or Streptococcus pneumoniae/ or exp Pneumococcal Infections/ or Pseudomonas/ or Pseudomonas aeruginosa/ or Acinetobacter/ or Acinetobacter baumannii/ or Acinetobacter Infections/ or Gram-Negative Bacteria/ or Gram-Positive Bacteria/ or Infection Control/ or Epidemics/ or Disease Outbreaks/) and Drug Resistance/) or Carbapenem-Resistant Enterobacteriaceae/

(((anti?microb* or anti?bacter* or bacter* or antibiotic* or microbial or (bacteri* adj4 (agent? or compound?)) or epidemic? or outbreak? or drug? or e-coli or streptococc* or pneumococc* or (staphylococc* adj2 aureus) or klebsiella or enterobacter* or pseudo?mona? or acinetobacter or gram-positive? or gram-negative? or (infect* adj4 control)) adj4 (resistan* or susceptib* or non?susceptib*)) or methicillin-resistant-staphylococcus-aureus or carbapenem-resistant-enterobacteri* or vancomycin-resistant-enterococc* or extended-spectrum-beta-lactam* or ((MRSA or CRE or VRE or ESBL) adj4 (infect* or bacter* or strain? or resistan* or isolat* or prevalence or incidence or drug?)) or (bacterial adj4 sensitivit*) or ((beta-lactam* or b-lactam* or carbapenem? or imipenem? or doripenem? or ertapenem? or meropenem? or cephalosporin? or cefazolin? or cefepim? or cefuroxim? or cefotaxim? or ceftriaxone? or ceftazidim? or cefoxitin? or ceftarolin? or ceftobiprol? or mono?bactam? or aztreonam? or penicillin? or ampicillin? or methicillin? or sulbactam? or tazobactam? or chloramphenicol? or tetracycline? or dox?cyclin? or tigec?clin? or trimethoprim? or vancomycin? or amino?glycoside? or gentam?cin? or kanam?cin? or fluoro?quinolone? or cipro?floxacin? or levo?floxacin? moxi?floxacin? or macrolide? or azithrom?cin?) adj3 resistan*)).mp.

or/1-3

Socioeconomic Factors/ or exp Economics, Hospital/ or exp Economics, Medical/ or Economics, Pharmaceutical/ or Health Care Sector/ or "Quality of Health Care"/ or Health Facilities/ or Health Services/ or Health Workforce/ or "Costs and Cost Analysis"/ or Cost-Benefit Analysis/ or Cost of Illness/ or Health Care Costs/ or Health Expenditures/ or "Quality of Life"/ or Hospitalization/ or Length of Stay/ or Quality-Adjusted Life Years/ or Mortality/ or Fatal Outcome/ or Death/ or Hospital Mortality/ or exp Morbidity/

((economic* adj4 (hospital? or medic* or pharma* or evaluat*)) or ((hospital* or medical*) adj4 (fee? or cost? or charge?)) or (pharma* adj4 audit?) or pricing or price? or burden or (cost* adj4 (illness or sickness or disease? or treatment? or data or compar* or minimi?ation or benefit? or utilit* or effective* or analys* or marginal? or out?of?pocket)) or (health adj3 (care or sector? or industr* or market? or qualit* or access* or evaluat* or facilit* or service? or workforce or manpower or man-power or cost?)) or ((expense? or expenditure? or payment? or spending) adj3 (health* or direct or indirect or out?of?pocket)) or affordabilit* or socio-economic* or (quality adj2 li?e*) or hospitali?ation* or (hospital adj stay*) or mortalit* or fatal* or death? or morbidit* or inciden* or prevalen* or hrqol or qol or qaly or daly or (quality adj adjusted adj life adj year*) or (disability adj adjusted adj life adj year*)).mp.

5 or 6

Armed Conflicts/ or Warfare/ or "Warfare and Armed Conflicts"/ or Afghan Campaign 2001-/ or Gulf War/ or Iraq War, 2003-2011/ or Vietnam Conflict/ or exp "Wounds and Injuries"/ or Wounds, Gunshot/ or Military Personnel/ or Developing Countries/ or Refugees/ or Vulnerable Populations/ or Human Migration/ or "Transients and Migrants"/ or Refugee Camps/ or Refugium/

(((conflict* or combat) adj2 (area? or zone? or setting* or region* or military or armed or ethnic or countr* or state? or field? or recovery)) or war* or battle* or battle?field* or combat* or incursion* or insurrection* or injur* or wound* or trauma* or migrant* or (asylum adj2 seeker*) or refugee* or displace* or (displace* adj2 (population* or people or person*))).mp.

8 or 9

Developing Countries.sh,kf.

((developing or less* developed or under developed or underdeveloped or middle income or low* income or underserved or under served or deprived or poor*) adj (countr* or nation? or population? or world)).ti,ab.

((developing or less* developed or under developed or underdeveloped or middle income or low* income) adj (economy or economies)).ti,ab.

(low adj3 middle adj3 countr*).ti,ab.

(lmic or lmics or third world or lmi countr*).ti,ab.

Afghanistan/ or Africa, Northern/ or Africa, Central/ or Algeria/ or Angola/ or Arabs/ or Asia, Central/ or Africa, Southern/ or Asia, Western/ or Baltic States/ or Brazil/ or "Bosnia and Herzegovina"/ or Burkina faso/ or Burundi/ or Cameroon/ or Chad/ or Colombia/ or Comoros/ or Cote d'Ivoire/ or Croatia/ or Democratic Republic of the Congo/ or Djibouti/ or Ecuador/ or Egypt/ or Eritrea/ or Ethiopia/ or Europe, Eastern/ or Gabon/ or Gambia/ or Ghana/ or Guinea/ or Guinea-Bissau/ or Haiti/ or India/ or Iran/ or Iraq/ or Jordan/ or Kenya/ or Kosovo/ or Kuwait/ or Lebanon/ or Liberia/ or Libya/ or Malawi/ or Mali/ or Mexico/ or Micronesia/ or Middle East/ or Montenegro/ or Morocco/ or Mozambique/ or Myanmar/ or Namibia/ or Niger/ or Nigeria/ or Pakistan/ or Papua New Guinea/ or Peru/ or Qatar/ or Rwanda/ or Saudi Arabia/ or Senegal/ or Serbia/ or Sierra Leone/ or Slovenia/ or Somalia/ or South Africa/ or South America/ or South Sudan/ or Sudan/ or Syria/ or Tajikistan/ or Tanzania/ or Timor-Leste/ or Tunisia/ or Turkey/ or Uganda/ or Uzbekistan/ or Venezuela/ or Yemen/ or Yugoslavia/ or Zimbabwe/

(afghan* or algeria* or angol* or Baltic? or bra?il* or bosnia* or her?egovina* or burkina fas?o or burundi* or chad* or colombia* or congo or (republic adj2 congo) or cote d?ivoire or ivory coast or croatia* or djibouti* or ecuador* or egypt* or eritrea* or ethiopia* or gabon* or ghana* or guinea or guinea-bissau or haiti* or india* or iran* or iraq* or jordan* or kenya* or kosovo or kurdistan* or kuwait* or kuweit* or liberia* or lebanon or liban or lebanese or libanaise or libya* or malawi* or mali* or mexic* or montenegro* or morocco or moroccan* or namibia* or niger* or pakistan* or palestin* or gaza or (west* adj2 bank) or peru* or qatar* or katar* or quatar* or rwanda* or ruanda* or saudi* or KSA or senegal* or serbia* or sierra leone or slovenia* or syria* or sudan* or somali* or tajikistan* or tanzania* or tunis* or turkey or uganda* or uzbekistan* or venezuela* or viet?nam* or yemen* or yugoslavia* or zimbabw* or MENA or EMRO or ((middle or near) adj2 east*) or (east* adj2 mediterranean) or orient or arabs or arab or arabia or levant or (east* adj2 europe*) or ((east* or north* or south* or centr*) adj2 africa*)). hw,kf,ti,ab,cp.

Military Personnel/ or Developing Countries/ or Refugees/ or Vulnerable Populations/ or Human Migration/ or "Transients and Migrants"/ or Refugee Camps/ or Refugium/

militar* or veteran* or migrant* or (asylum adj2 seeker*) or refugee* or displace* or (displace* adj2 (population* or people or person*)).mp.

or/11-19

4 and 7 and 10 and 20

limit 21 to yr="1990-current"

**Appendix III.** Categorization of countries according to the World Bank Data

**Fragile and conflict affected situations**

Afghanistan, Burkina Faso, Burundi, Cameroon, Central African Republic, Chad, Comoros, Democratic Republic of Congo, Republic of Congo, Eritrea, The Gambia, Guinea-Bissau, Haiti, Iraq, Kiribati, Kosovo, Lebanon, Liberia, Libya, Mali, Marshall Islands, Federated States of Micronesia, Myanmar, Niger, Nigeria, Papua New Guinea, Solomon Islands, Somalia, South Sudan, Sudan, Syrian Arab Republic, Timor-Leste, Tuvalu, Bolivarian Republic of Venezuela, West Bank and Gaza, Republic of Yemen, Zimbabwe

**Low & middle income**

Afghanistan, Albania, Algeria, American Samoa, Angola, Argentina, Armenia, Azerbaijan, Bangladesh, Belarus, Belize, Benin, Bhutan, Bolivia, Bosnia and Herzegovina, Botswana, Brazil, Bulgaria, Burkina Faso, Burundi, Cabo Verde, Cambodia, Cameroon, Central African Republic, Chad, China, Colombia, Comoros, Democratic Republic of Congo, Republic of Congo, Costa Rica, Cote d’Ivoire, Cuba, Djibouti, Dominica, Dominican Republic, Ecuador, Arab Republic of Egypt, El Salvador, Equatorial Guinea, Eritrea, Eswatini, Ethiopia, Fiji, Gabon, The Gambia, Georgia, Ghana, Grenada, Guatemala, Guinea, Guinea-Bissau, Guyana, Haiti, Honduras, India, Indonesia, Islamic Republic of Iran, Iraq, Jamaica, Jordan, Kazakhstan, Kenya, Kiribati, Democratic People’s Republic of Korea, Kosovo, Kyrgyz Republic, Lao PDR, Lebanon, Lesotho, Liberia, Libya, Madagascar, Malawi, Malaysia, Maldives, Mali, Marshall Islands, Mauritania, Mexico, Federated States of Micronesia, Moldova, Mongolia, Montenegro, Mozambique, Myanmar, Namibia, Nepal, Nicaragua, Niger, Nigeria, North Macedonia, Pakistan, Papua New Guinea, Paraguay, Peru, Philippines, Russian Federation, Rwanda, Samoa, Sao Tome and Principe, Senegal, Serbia, Sierra Leone, Solomon Islands, Somalia, South Africa, South Sudan, Sri Lanka, St. Lucia, St. Vincent and the Grenadines, Sudan, Suriname, Syrian Arab Republic, Tajikistan, Tanzania, Thailand, Timor-Leste, Togo, Tonga, Tunisia, Turkey, Turkmenistan, Tuvalu, Uganda, Ukraine, Uzbekistan, Vanuatu, Bolivarian Republic of Venezuela, Vietnam, West Bank and Gaza, Republic of Yemen, Zambia, Zimbabwe

**Appendix IV. a.** Quality assessment checklist for nonrandomized studies (excluding cohort studies).

| **Domain** | **Checklist Criteria** | **Additional Information** |
| --- | --- | --- |
| Selection | **1) Is the case definition adequate?**  a) yes, with independent validation *****  b) yes, eg record linkage or based on self-reports  c) no description | Assumption: Retrospective chart review studies received one star if the case was defined according to health records. |
|  | **2) Representativeness of the cases**  a) consecutive or obviously representative series of cases *  b) potential for selection biases or not stated | Assumption: Given the nature of the review, studies received a star if they discuss representation (and reasons for their study being representative) or if they are multicentre/regional/national. |
|  | **3) Selection of Controls**  a) community controls *****  b) hospital controls  c) no description | Assumption: It was assumed that if the study was in a hospital setting in which cases were hospital patients, hospital controls were accepted. |
|  | **4) Definition of Controls**  a) no history of disease (endpoint) *****  b) no description of source | Assumption: History of disease/infection was used in this criteria even in studies looking at mortality or other burden outcomes |
| Comparability | **1) Comparability of cases and controls on the basis of the design or analysis**  a) study controls for age/sex/comorbidities *  b) study controls for any additional factor ***** |  |
| Exposure | **1) Ascertainment of exposure**  a) secure record (eg surgical records) *****  b) structured interview where blind to case/control status *****  c) interview not blinded to case/control status  d) written self-report or medical record only  e) no description | Assumption: Studies which utilised lab techniques to ascertain exposure received one star. |
|  | **2) Same method of ascertainment for cases and controls**  a) yes *****  b) no |  |
|  | **3) Non-Response rate**  a) same rate for both groups *****  b) non respondents described  c) rate different and no designation | Assumption: No description of data cleaning or linkage and loss to missing data for retrospective studies was panelised by not awarding a star |

**Appendix IV. b.** Quality assessment checklist for cohort studies.

| **Domain** | **Checklist Criteria** |
| --- | --- |
| Selection | **1) Representativeness of the exposed cohort**  a) truly representative of the average _______________ (describe) in the community *  b) somewhat representative of the average ______________ in the community *  c) selected group of users eg nurses, volunteers  d) no description of the derivation of the cohort |
|  | **2) Selection of the non-exposed cohort**  a) drawn from the same community as the exposed cohort *  b) drawn from a different source  c) no description of the derivation of the non-exposed cohort |
|  | **3) Ascertainment of exposure**  a) secure record (eg surgical records) *  b) structured interview *  c) written self-report  d) no description |
|  | **4) Demonstration that outcome of interest was not present at start of study**  a) yes *  b) no |
| Comparability | **1) Comparability of cases and controls on the basis of the design or analysis**  a) study controls for age/sex/comorbidities *  b) study controls for any additional factor * |
| Outcome | **1) Assessment of outcome**  a) independent blind assessment *  b) record linkage *  c) self-report  d) no description |
|  | **2) Was follow-up long enough for outcomes to occur**  a) yes (select an adequate follow up period for outcome of interest) *  b) no |
|  | **3) Adequacy of follow up of cohorts**  a) complete follow up - all subjects accounted for *  b) subjects lost to follow up unlikely to introduce bias - small number lost - > ____ % (select an  adequate %) follow up, or description provided of those lost) *  c) follow up rate < ____% (select an adequate %) and no description of those lost  d) no statement |
